# Tracheotomy in Scarlet Fever

**Published:** 1899-07-29

**Authors:** 


### TRACHEOTOMY IN SCARLET FEVER.

In the medical supplement to the recently issued annual report of the Metropolitan Asylums Board there is an interesting paper by Dr. Stewart, assistant medical officer, South-Western Hospital, on "The Value of Tracheotomy in Certain Cases of Septic Scarlet Fever," a paper in which, as it seems to us, he opens up new ground. The condition of obstructed breathing which occurs in the cases alluded to is exclusively due to faucio-nasal obstruction, and is not in any way connected with laryngeal swelling, It is a condition which is present in a large number of the fatal cases of septic scarlet fever, and occurs almost entirely in young children. The respiratory difficulty may not be in itself the actual cause of death, but it conduces to a fatal issue by depriving the child of sleep. It is distinctive of this kind of obstruction that it only becomes obvious when the child is falling off to sleep, whereas that due to laryngeal obstruction is apparent whether the child is asleep or awake. The character of the respiratory distress is very distinctive. Under normal circumstances a child on falling asleep breathes through his nose, but in this condition, owing to the complete blocking of the nasal passages, he utterly fails to do so. At the same time, he is unable to breathe through the mouth owing to the degree of faucial swelling, and to some extent to the falling back of the tongue. The admission of air being thus impeded; retraction of the chest wall takes place, just as in laryngeal obstruction, the child becomes cyanosed, and is wakened up with a sense of suffocation. After three or four respirations, which quickly restore the colour, the patient falls off to sleep again, only, however, to repeat the same performance, being forced to wake every three or four minutes to fill his lungs, thus practically getting no rest at all. Relief was fully afforded by tracheotomy, the patients falling to sleep almost immediately. The operation, however, must be undertaken with a full sense of its gravity, for in the cases mentioned the wounds by no means did well. The edges became inflamed, and the deeper parts dry and void of granulations. Moreover, the cellular tissue and fascia between the muscles in the immediate neighbourhood sloughed away, and in all the cases there was considerable difficulty in dispensing with the tube, thus contrasting strongly with what obtains after tracheotomy in laryngeal diphtheria. Of the four cases in which the operation was performed for the relief of this serious condition, three recovered and one died. One of the cases which recovered was still wearing the tube when it was discharged, six months after the operation. It must be remembered that the condition is one of immense gravity, and we feel quite clear that where the dyspnœa cannot be overcome by attention to the position of the patient, and is sufficiently severe to produce cyanosis and retraction of the ribs, there is but little chance of recovery without operation, and that the performance of tracheotomy in such cases as suggested by Dr. Stewart is a distinct advance.

